# Supplementary material for: Telomere length and dynamics in Astyanax mexicanus cave and surface morphs
Source: PeerJ. 2024 Feb 28;12:e16957. doi: 10.7717/peerj.16957 (PMC10908260; doi:10.7717/peerj.16957)
Supplement: Supplemental Information 5 — DNA concentrations are expressed in ng/ μl, while each sample refers to a different fish. [file peerj-12-16957-s005.docx]

**Table S4 The average Ct values from DNA serial dilution test**

The average of Ct values from the serial dilution test aiming to assess the working concentration of DNA. DNA concentrations are expressed in ng/µl, while each sample refers to a different fish.

|  | **DNA concentrations** | | | |
| --- | --- | --- | --- | --- |
| **Samples** | 9 | 0.9 | 0.09 | 0.009 |
| Sample 1 | **14.31** | 17.27 | 20.79 | 23.96 |
| Sample 2 | **14.06** | 16.61 | 21.06 | 24.07 |
| Sample 3 | 17.04 | 20.02 | 24.23 | 26.87 |
| Sample 4 | **14.74** | 17.61 | 21.25 | 24.30 |
| Sample 5 | 16.65 | 19.75 | 23.79 | 26.68 |
