# Supplementary material for: Telomere length and dynamics in Astyanax mexicanus cave and surface morphs
Source: PeerJ. 2024 Feb 28;12:e16957. doi: 10.7717/peerj.16957 (PMC10908260; doi:10.7717/peerj.16957)
Supplement: Supplemental Information 6 — Minimum Information for Publication of Quantitative Real-Time PCR Experiments checklist. [file peerj-12-16957-s006.docx]

Table 1. MIQE checklist for authors, reviewers, and editors.

| Item to check | Importance | Reported | Item to check | Importance |  |
| --- | --- | --- | --- | --- | --- |
| Experimental design | | | qPCR oligonucleotides | |  |
| Definition of experimental and control groups | E | Lines 101-103 | Primer sequences | E | Lines 162-166 |
| Number within each group | E | Lines 116-117, 127-128, 137-138 | RTPrimerDB identification number | D | NA |
| Assay carried out by the core or investigator’s laboratory? | D | NA | Probe sequences | D | NA |
| Acknowledgment of authors’ contributions | D | On line submission system | Location and identity of any modifications | E | NA |
| Sample | | | Manufacturer of oligonucleotides | D | N |
| Description | E | Lines 101-103, 116-117, 127-128, 137-138 | Purification method | D | N |
| Volume/mass of sample processed | D | NA | qPCR protocol | | |
| Microdissection or macrodissection | E | Line 129 | Complete reaction conditions | E | Lines 174-180 |
| Processing procedure | E | Lines 127-130, 137-144 | Reaction volume and amount of cDNA/DNA | E | Lines 174-176 |
| If frozen, how and how quickly? | E | NA | Primer, (probe), Mg2, and dNTP concentrations | E | Lines 174-175 |
| If fixed, with what and how quickly? | E | NA | Polymerase identity and concentration | E | Lines 174-175 |
| Sample storage conditions and duration (especially for FFPEb samples) | E | NA | Buffer/kit identity and manufacturer | E | Lines 174-175 |
| Nucleic acid extraction | | | Exact chemical composition of the buffer | D | Lines 174-175 |
| Procedure and/or instrumentation | E | Lines 146-151 | Additives (SYBR Green I, DMSO, and so forth) | E | Lines 174-175 |
| Name of kit and details of any modifications | E | Line 149 | Manufacturer of plates/tubes and catalog number | D | N |
| Source of additional reagents used | D | Lines 147-151 | Complete thermocycling parameters | E | Lines 178-179 |
| Details of DNase or RNase treatment | E | NA | Reaction setup (manual/robotic) | D | N |
| Contamination assessment (DNA or RNA) | E | NA | Manufacturer of qPCR instrument | E | Line 161 |
| Nucleic acid quantification | E | Lines 149-151 | qPCR validation | | |
| Instrument and method | E | Lines 149-151 | Evidence of optimization (from gradients) | D | N |
| Purity (A260/A280) | D | N | Specificity (gel, sequence, melt, or digest) | E | Lines 169-170 |
| Yield | D | N | For SYBR Green I, Cq of the NTC | E | Lines 172-175 |
| RNA integrity: method/instrument | E | NA | Calibration curves with slope and y intercept | E | Lines 170-174 |
| RIN/RQI or Cq of 3 and 5 transcripts | E | NA | PCR efficiency calculated from slope | E | Lines 173-174 |
| Electrophoresis traces | D | N | CIs for PCR efficiency or SE | D | N |
| Inhibition testing (Cq dilutions, spike, or other) | E | N | r2 of calibration curve | E | Lines 172-175 |
| Reverse transcription | |  | Linear dynamic range | E | N |
| Complete reaction conditions | E | NA | Cq variation at LOD | E | Lines 183-186 |
| Amount of RNA and reaction volume | E | NA | CIs throughout range | D | N |
| Priming oligonucleotide (if using GSP) and concentration | E | NA | Evidence for LOD | E | Lines 153-161 |
| Reverse transcriptase and concentration | E | NA | If multiplex, efficiency and LOD of each assay | E | NA |
| Temperature and time | E | NA | Data analysis | | |
| Manufacturer of reagents and catalogue numbers | D | NA | qPCR analysis program (source, version) | E | Line 186 |
| Cqs with and without reverse transcription | D | NA | Method of Cq determination | E | Line 186 |
| Storage conditions of cDNA | D | NA | Outlier identification and disposition | E | Lines 189-191 |
| qPCR target information | | | Results for NTCs | E | Lines 190-192 |
| Gene symbol | E | Line 166 | Justification of number and choice of reference genes | E | Lines 166-168 |
| Sequence accession number | E | Line 166 | Description of normalization method | E | Lines 192-194 |
| Location of amplicon | D | Line 169 | Number and concordance of biological replicates | D | N |
| Amplicon length | E | Line 169 | Number and stage (reverse transcription or qPCR) of technical replicates | E | Line 177 |
| In silico specificity screen (BLAST, and so on) | E | Line 169-170 | Repeatability (intraassay variation) | E | Lines 180-183 |
| Pseudogenes, retropseudogenes, or other homologs? | D | N | Reproducibility (interassay variation, CV) | D | Lines 183-186 |
| Sequence alignment | D | N | Power analysis | D | N |
| Secondary structure analysis of amplicon | D | N | Statistical methods for results significance | E | Lines 201-224 |
| Location of each primer by exon or intron (if applicable) | E | Line 169 | Software (source, version) | E | Lines 197-199 |
| What splice variants are targeted? | E | NA | Cq or raw data submission with RDML | D | N |
